# Rapid development of Purkinje cell excitability, functional cerebellar circuit, and afferent sensory input to cerebellum in zebrafish

**DOI:** 10.3389/fncir.2014.00147

**Published:** 2014-12-19

**Authors:** Jui-Yi Hsieh, Brittany Ulrich, Fadi A. Issa, Jijun Wan, Diane M. Papazian

**Affiliations:** ^1^Department of Physiology, David Geffen School of Medicine at University of California Los AngelesLos Angeles, CA, USA; ^2^Interdepartmental Ph.D. Program in Molecular, Cellular, and Integrative Physiology, David Geffen School of Medicine at University of California Los AngelesLos Angeles, CA, USA; ^3^Department of Neurology, David Geffen School of Medicine at University of California Los AngelesLos Angeles, CA, USA; ^4^Molecular Biology Institute, David Geffen School of Medicine at University of California Los AngelesLos Angeles, CA, USA

**Keywords:** Purkinje cell, patch clamp, cerebellum, zebrafish, parallel fiber, climbing fiber, visual input

## Abstract

The zebrafish has significant advantages for studying the morphological development of the brain. However, little is known about the functional development of the zebrafish brain. We used patch clamp electrophysiology in live animals to investigate the emergence of excitability in cerebellar Purkinje cells, functional maturation of the cerebellar circuit, and establishment of sensory input to the cerebellum. Purkinje cells are born at 3 days post-fertilization (dpf). By 4 dpf, Purkinje cells spontaneously fired action potentials in an irregular pattern. By 5 dpf, the frequency and regularity of tonic firing had increased significantly and most cells fired complex spikes in response to climbing fiber activation. Our data suggest that, as in mammals, Purkinje cells are initially innervated by multiple climbing fibers that are winnowed to a single input. To probe the development of functional sensory input to the cerebellum, we investigated the response of Purkinje cells to a visual stimulus consisting of a rapid change in light intensity. At 4 dpf, sudden darkness increased the rate of tonic firing, suggesting that afferent pathways carrying visual information are already active by this stage. By 5 dpf, visual stimuli also activated climbing fibers, increasing the frequency of complex spiking. Our results indicate that the electrical properties of zebrafish and mammalian Purkinje cells are highly conserved and suggest that the same ion channels, Nav1.6 and Kv3.3, underlie spontaneous pacemaking activity. Interestingly, functional development of the cerebellum is temporally correlated with the emergence of complex, visually-guided behaviors such as prey capture. Because of the rapid formation of an electrically-active cerebellum, optical transparency, and ease of genetic manipulation, the zebrafish has great potential for functionally mapping cerebellar afferent and efferent pathways and for investigating cerebellar control of motor behavior.

## Introduction

The zebrafish, a lower vertebrate, has great potential for optogenetic mapping of brain circuits because of its transparency, rapid development, and ease of genetic manipulation (Arrenberg and Driever, 2013). The power of optogenetic mapping is increased significantly by the ability to record electrophysiologically in live animals from neurons that integrate converging input information and generate output signals for efferent pathways. One such neuron is the cerebellar Purkinje cell, the sole output neuron of the cerebellar cortex. Little is known about the electrical activity of cerebellar neurons in zebrafish. We have now used patch clamp electrophysiology *in situ* in live zebrafish to investigate the electrical properties of Purkinje cells, the functional maturation of the cerebellar circuit, and the emergence of sensory input to the cerebellum during brain development.

The embryological origins and anatomical organization of cerebellar neurons are highly conserved in zebrafish and mammals (Hashimoto and Hibi, 2012). Advantageously for brain mapping, the cerebellum is smaller, simpler, and develops much more rapidly in zebrafish than in mammals. The zebrafish cerebellum has three lobes, the corpus cerebelli (CCe), the valvula cerebelli (Va), and the vestibulolateral lobe. CCe and Va have tri-lamellar structures comprising the granule cell, Purkinje cell, and molecular layers. These layers have the same orientation in CCe as in the mammalian cerebellum, but are inverted in Va (Bae et al., 2009; Hashimoto and Hibi, 2012). In contrast, the vestibulolateral lobe contains only the granule cell layer (Hashimoto and Hibi, 2012). Thus, the CCe lobe of the zebrafish cerebellum has the strongest similarity to the mammalian cerebellum. In mammals, Purkinje cell compartments have been defined by stripes of aldolase-C (zebrin-II) expression (Ji and Hawkes, 1994). In contrast, it has been shown that zebrin-II is expressed specifically and exclusively in all zebrafish Purkinje cells (Bae et al., 2009). Anatomical evidence suggests that zebrafish Purkinje cells, like their mammalian counterparts, receive two types of direct excitatory inputs, parallel fibers and climbing fibers, which are the axons of cerebellar granule cells and inferior olive neurons, respectively (Bae et al., 2009; Hashimoto and Hibi, 2012). In mammals, parallel fibers convey sensory and predictive information that is carried into the cerebellum by mossy fibers from precerebellar nuclei, whereas climbing fibers provide error correction signals that help to optimize motor control (D’Angelo et al., 2011). The interaction between parallel fiber and climbing fiber inputs to Purkinje cells is crucial for motor learning (Ito, 2002a,b, 2006). The function of parallel and climbing fiber inputs in zebrafish has not yet been investigated. Zebrafish Purkinje cells target eurydendroid cells, which are equivalent to deep cerebellar nuclei neurons in mammals (Hibi and Shimizu, 2012). Similar to deep cerebellar nuclei neurons, eurydendroid cells project to the hindbrain, tectum, and thalamus (Heap et al., 2013).

We found that zebrafish Purkinje cells, which are born at 3 days post-fertilization (dpf; Bae et al., 2009), are electrically excitable by 4 dpf. A mature pattern of spontaneous tonic firing interspersed with complex spiking is established over the next 48 h. The electrical properties and the expression of ion channels that control firing are highly conserved compared to mammalian Purkinje cells. By 4 dpf, Purkinje neurons receive visual input conveyed by mossy fibers to parallel fibers, with visual input via climbing fibers developing by the next day. Rapid development of a functional cerebellum is likely to be an essential survival advantage in zebrafish, which develop entirely outside the body of the mother where they must avoid predators and find food starting early in life. A notable advantage of zebrafish is that electrophysiological analysis of Purkinje cells is technically easy in a minimally-disturbed, live animal preparation with intact brain, sensory input, and motor output. This facilitates experiments that would be technically challenging and significantly more invasive in mammals. Our results indicate that the zebrafish cerebellum is an excellent system in which to combine optogenetic mapping and electrophysiological analysis, to evaluate emerging optical methods for functional brain mapping, and to investigate cerebellar control of motor behavior and motor learning.

## Materials and methods

### Animal maintenance

Zebrafish (*Danio rerio*) were housed in the University of California, Los Angeles (UCLA) Zebrafish Core Facility at 28°C using a 14 h/10 h light/dark cycle. Adults were bred to obtain embryos. Animals were raised until 9 dpf in a 28°C incubator using the same light/dark cycle. Starting at 5 dpf, larvae were fed brine shrimp powder twice daily. Animal procedures were approved by the Chancellor’s Animal Research Committee at UCLA.

### Generation of transgenic zebrafish

Previously, a zebrafish transgenic line, rk22Tg:*Tg(aldoca:gap43-Venus)*, which expresses a membrane-bound form of the Venus yellow fluorescent protein specifically in cerebellar Purkinje cells under the control of the zebrafish *aldolase Ca* (*aldoca*) promoter, was generated in the AB wild type strain by Tanabe et al. (2010). Aldolase C (zebrin-II) is a specific Purkinje cell marker in zebrafish and mammals (Brochu et al., 1990; Tanabe et al., 2010). Membrane tethering of Venus is conferred by a palmitoylation site in the first 20 amino acids of zebrafish gap43, which have been fused to the Venus N-terminus. Using a plasmid containing the *aldoca*:gap43-Venus insert in the pT2K vector (kind gift of Dr. Masahiko Hibi), we generated an equivalent transgenic line, la118Tg:*Tg(aldoca:gap43-Venus)*, in the unpigmented Tüpfel long fin nacre (TLN) strain using Tol2-mediated transgenesis (Kawakami et al., 2004; Schoonheim et al., 2010; Tanabe et al., 2010,^1^). Plasmid DNA and *in vitro* transcribed transposase mRNA were mixed and co-injected into single-celled embryos (Kawakami et al., 2004). Injected fish were raised to sexual maturity and bred. Germline transgenic animals were identified in the F1 generation by gap43-Venus expression in Purkinje cells.

### RNA *in situ* hybridization

*In situ* hybridization was used to investigate Purkinje cell-specific expression of Kv3.3 and Nav1.6. Probes derived from the zebrafish *kcnc3a* (Kv3.3a) or *scn8aa* (Nav1.6a) genes were separately mixed with an *aldoca* (zebrin-II) probe, which was used to identify differentiated Purkinje cells (Tsai et al., 2001; Bae et al., 2009; Mock et al., 2010; Tanabe et al., 2010). Whole mount, double fluorescent *in situ* hybridization was performed as described by Brend and Holley (2009) at 3, 4, 5, and 6 dpf using TLN zebrafish. Animals were euthanized by immersion in 0.2% MS222 and then fixed overnight (~15 h) in 4% paraformaldehyde at 4°C. Yolk sacs were removed by dissection. Larvae were permeabilized by digesting with 200 μg/mL proteinase K for 25 min at room temperature. Zebrafish were incubated overnight at 68°C in 50 μL prehybridization buffer containing 1 μL *aldoca* probe and 1 μL *kcnc3a* or 1μL *scn8aa* probe. Fluorescein-labeled *kcnc3a* and *scn8aa* probes were visualized using the TSA Plus Fluorescein Kit (PerkinElmer) with an incubation time of 45 min at room temperature. Digoxigenin-labeled *aldoca* probes were visualized using the TSA Plus Cy5 Solution (PerkinElmer) with an incubation time of 30 min at room temperature. Specimens were mounted dorsal side up in 75% glycerol. Images were acquired using an Olympus Fluoview FV300 laser scanning confocal microscope with an Olympus 40×/1.3 oil immersion objective. Signals from antisense and sense probes were imaged using the same laser settings.

Gene-specific probes for *aldoca* and *scn8aa* were made using primers from the last coding exon and the 3′ untranslated region of each gene (Thisse and Thisse, 2008). To generate antisense and sense probes for the zebrafish *aldoca* gene (NM_001029952), genomic DNA was amplified by PCR using the forward primer 5′-ATTTAGGTGACACTATAGAAGGGAAGTACACGGTTTGTGGTGA-3′ and the reverse primer 5′-TAATACGACTCACTATAGGGCACATCTCACAGTTTTATTGCAGCAC-3′. The 698 bp product was transcribed using the Digoxigenin RNA Labeling Kit (Roche Diagnostics) by T7 RNA polymerase to make the antisense probe and SP6 RNA polymerase to make the sense probe. Probes to *scn8aa* (NM_131628) were amplified from zebrafish genomic DNA using the forward primer 5′-ATTTAGGTGACACTATAGAAGACAGTAAGGGCAAAAAGGGCAA-3′ and the reverse primer 5′-TAATACGACTCACTATAGGGAATGGGCTGAACGTTTTCCCC-3′. The 703 bp product was transcribed using the RNA Labeling Kit with Fluorescein NTP Labeling Mix (Roche Diagnostics) by T7 RNA polymerase to make the antisense probe and by SP6 RNA polymerase to make the sense probe. Probes for *kcnc3a* (NM_001195240.1) were made using a clone containing the first 1363 bp of the cDNA sequence in the pCRII vector (Mock et al., 2010). To generate the antisense probe, the clone was linearized by digestion with HindIII (New England BioLabs) and transcribed by T7 RNA polymerase using the RNA Labeling Kit with Fluorescein NTP Labeling Mix (Roche Diagnostics). For the sense probe, the clone was linearized with XhoI (New England BioLabs) and transcribed by SP6 RNA polymerase.

### *In situ* electrophysiology

Larval zebrafish between 4 and 14 dpf were anesthetized with medical grade 0.02% MS222 (Western Chemical) for ~10 s, then glued dorsal side up onto coverslips in a recording chamber. The chamber was filled with external solution containing (in mM): 134 NaCl, 2.9 KCl, 2.1 CaCl_2_, 1.2 MgCl_2_, 10 HEPES, and 10 glucose, pH 7.5. Curare (10 μM) was added to paralyze the animals. Skin around the head and the skull were gently removed using fine forceps. Electrophysiological recordings were performed in awake animals starting 5–10 min after the dissection and were stable for up to 1 h. All data shown in this study were acquired within 45 min after the dissection. To avoid possible circadian variation in the results, all recordings were made between noon and 6 pm local time. At the end of the experiment, zebrafish were euthanized by immersion in 0.2% MS222.

Data were acquired in the cell-attached, loose patch configuration using a HEKA EPC10 patch clamp amplifier and Pulse software (HEKA Elektronik). Borosilicate pipettes (7–10 MΩ, World Precision 1B150F-4) were filled with external solution. Purkinje cells were visualized under an upright Olympus BX51WI microscope using a 40×/0.80 water-immersion lens. Recordings were made from cells in the CCe. Patch pipettes approached Purkinje cells from the rostral side at an angle of 30° relative to the horizontal plane. Seal resistances ranged from 20 MΩ to 2 GΩ. Experiments were performed at room temperature (22–25°C) with the ambient and microscope lights turned off. Electrical activity was recorded in voltage clamp mode at 0 mV. Data were acquired at 20–50 kHz and filtered at 3 kHz. Only recordings with consistent spike amplitudes and non-overlapping spike waveforms were analyzed.

### Inferior olive stimulation

Inferior olive neurons were stimulated using a theta pipette (~5 μm tip opening) filled with external solution. Depending on the age of the animal, the tip of the theta pipette was placed 250–300 μm anterior to the end of brainstem at a depth of 125–150 μm from the dorsal surface. These coordinates were chosen based on a previous anatomical study by Bae et al. (2009) and the results of our preliminary experiments. To identify an appropriate stimulus amplitude, 2 ms pulses ranging from 20 to 200 μA were delivered to the inferior olive using a stimulus isolator (A.M.P.I.). A stimulus of 50 μA evoked maximal complex spiking in Purkinje cells by the criterion that there was no apparent difference in the response when larger stimuli were applied. Therefore, a 2 ms 50 μA stimulus was used in all experiments. Data were analyzed only from Purkinje cells that showed complex spiking in the absence of direct olivary stimulation. Otherwise, if stimulation failed to elicit complex spikes, we could not distinguish the scenario in which there was no climbing fiber-Purkinje cell connection from the scenario in which inferior olive neurons were damaged by the placement of the theta pipette.

### Visual stimulation experiments

A 1 W, 6500K white LED light source (Thorlabs) was installed on the microscope to illuminate the preparation through the halogen light pathway. A white LED was chosen to avoid possible color-dependent variations in Purkinje cell responses. Animals were light adapted for 2 min before the LED was turned off. In all experiments, light adaptation was performed using the minimum LED intensity that evoked consistent Purkinje cell responses. The power of the LED was controlled by the HEKA EPC10 amplifier. Switching the LED on and off was time-locked with electrophysiological recordings. Purkinje cell activity was recorded for 10 s before and 10–60 s after the LED was turned off. To avoid over-stimulating the visual system, recordings were made from one Purkinje cell per animal in most cases. Occasionally, recordings were made from two Purkinje cells in the same animal. Each Purkinje cell was subjected to 1–4 trials, with an inter-trial interval of at least 2 min. No signs of habituation or desensitization were noted when multiple trials were performed on the same cell.

### Data analysis

Electrophysiological data were imported into Igor 6.2 (WaveMetrics) and analyzed with Clampfit 10.2 and 10.4 (Molecular Devices). The regularity of spontaneous tonic firing was quantified by determining the coefficient of variation of adjacent intervals (CV2) defined as, $\frac{2}{n}\sum_{n}^{n}\frac{\left|I_{i+1}-I_{i}\right|}{I_{i+1}+I_{i}}$ where *I* is interspike interval in ms. CV2 is preferable to the conventional coefficient of variation (CV, standard deviation/mean) for data recorded on a short time scale (Walter et al., 2006; Wulff et al., 2009). Data are provided as mean ± SEM. Statistical significance was assessed using ANOVA followed by either Tukey’s or Holm-Bonferroni *post hoc* tests. Spearman’s rank method was used for correlation analysis. Excel 2011 (Microsoft) and Origin 8 (OriginLab) were used to perform statistical testing. Adobe Illustrator (Adobe) was used to prepare figures.

## Results

### Rapid emergence of electrical excitability in zebrafish Purkinje cells

**Figure 1:**
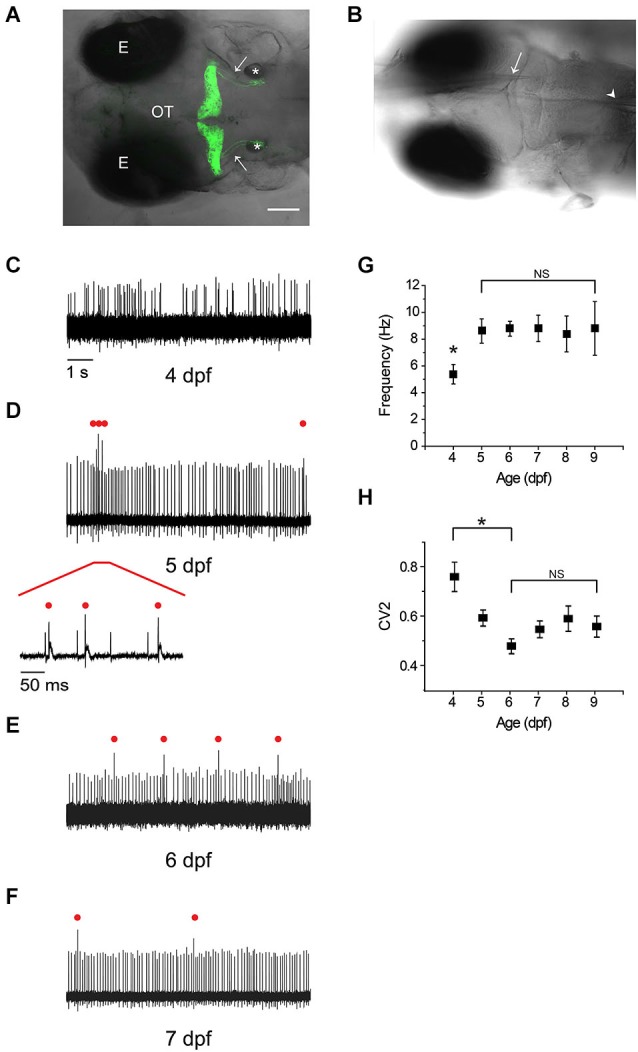
**Rapid emergence and maturation of excitability in zebrafish Purkinje cells. (A)** Dorsal view of la118Tg:*Tg(aldoca:gap43-Venus)* transgenic zebrafish at 5 dpf shows that membrane-bound Venus is specifically and exclusively expressed in cerebellar Purkinje cells (Tanabe et al., 2010). Anterior is to the left. A z-projection of 168 1 μm confocal images has been superimposed on the corresponding bright field image. Images were acquired with an Olympus Fluoview FV300 laser scanning confocal microscope. Labels: E, eye; OT, optic tectum; *, otic vesicle; arrows, cerebellovestibular axon tracts. Scale bar, 100 μm. **(B)** The recording configuration is shown. A transgenic zebrafish at 5 dpf has been fixed in the recording chamber. A loose-patch electrode (arrow) was inserted from the anterior side and advanced to contact the cell body of a Purkinje cell. In some experiments, a theta pipette (arrowhead) was placed in the inferior olive to stimulate climbing fibers. **(C–F)** Shown are representative loose-patch recordings acquired at **(C)** 4 dpf, **(D)** 5 dpf, **(E)** 6 dpf, and **(F)** 7 dpf. In **(D)**, red bar marks portion of trace that is shown on an expanded time scale below the main trace. In **(D)**, **(E)**, and **(F)**, complex spike-like events are indicated by red dots. **(G)** The average simple spike firing frequency has been plotted vs. age (*n* = 7–31 cells from 6–24 animals). The frequency at 4 dpf, 5.4 Hz, was significantly different from the frequency on all subsequent days, which averaged ~9 Hz (*, ANOVA: 4–9 dpf, *F*_(5,113)_ = 2.41, *p* = 0.04, followed by Tukey’s *post hoc* test: 4 and 5 dpf, *p* < 0.05). Values obtained between 5 and 9 dpf did not differ significantly (NS, ANOVA: 4–9 dpf, *F*_(5,113)_ = 2.41, *p* = 0.04, followed by Holm-Bonferroni *post hoc* test: 5–9 dpf, *p* ≥ 0.05). **(H)** The regularity of spontaneous tonic firing was quantified by determining the coefficient of variation of adjacent intervals (CV2), which has been plotted against age(*n* = 7–31 cells from 6–24 animals). CV2 declined significantly from 4 dpf (CV2 = 0.76) to 6 dpf (CV2 = 0.48); CV2 did not differ significantly from 6–9 dpf (ANOVA: 4–9 dpf, *F*_(5,113)_ = 5.83, *p* = 7.96 × 10^−5^, followed by Holm-Bonferroni *post hoc* test: 4–6 dpf, *p* < 0.05 [*] and 6–9 dpf, *p* ≥ 0.05 [NS]).

During brain development in zebrafish, *ptf1a*-expressing precursor cells located in the ventricular zone give rise to cerebellar Purkinje cells which are born, differentiate, migrate, and begin to extend neurites at 3 dpf (Bae et al., 2009; Kani et al., 2010; Tanabe et al., 2010). The primary dendrite and axon form at 4 dpf, and fine branches and spines become visible shortly thereafter. Starting at 4 dpf, we used patch clamp electrophysiology to investigate the development of electrical excitability in Purkinje cells. Experiments were performed at room temperature (22–25°C) using a transgenic zebrafish line made in the unpigmented Tüpfel long fin nacre (TLN) strain that expresses a membrane-bound form of Venus, a yellow fluorescent protein, specifically in Purkinje cells under the control of the zebrafish *aldoca* promoter (Figure 1A; Schoonheim et al., 2010; Tanabe et al., 2010). After removing the overlying skin and skull, recordings were made using loose patch electrodes in live, awake animals *in situ* in the intact brain (Figure 1B). Recordings were made primarily from Purkinje cells located in the medial region of each cerebellar hemisphere.

At 4 dpf, Purkinje cells were already electrically excitable, with spontaneous, irregular firing of action potentials at an average frequency of ~5 Hz at room temperature (Figure 1C). By 5 dpf, the average tonic firing frequency had increased significantly to ~9 Hz, a value that did not change significantly on subsequent days (Figures 1D–G). For comparison, the tonic firing rate in mammalian Purkinje cells in acute cerebellar slices is ~12 Hz at room temperature (23–26°C) (Wulff et al., 2009).

The regularity of tonic firing was assessed by measuring the coefficient of variation of adjacent intervals (CV2). Firing regularity increased significantly between 4 and 6 dpf, as indicated by a decrease in CV2 from ~0.8 at 4 dpf to ~0.5 at 6 dpf (Figure 1H). In contrast, CV2 did not differ significantly from 6–9 dpf (Figure 1H). These values are higher than that reported for rodent Purkinje cells in cerebellar slices (~0.2) (Wulff et al., 2009). However, the numbers are not directly comparable because the animals in our experiments were awake and could have been responding to uncontrolled sensory stimuli, which would be expected to increase the variability of tonic firing. Consistent with this interpretation, CV2 for tonic firing is ~0.4–0.6 in awake mice, and the coefficient of variation of the interspike interval (CV, standard deviation/mean) is ~0.5–1 in awake cats (Armstrong and Rawson, 1979; Wulff et al., 2009; Zhou et al., 2014).

In addition to tonic spiking, by 5 dpf a majority of cells occasionally fired action potentials with more complex waveforms, consisting of a large initial spike followed by a longer lasting but lower amplitude depolarization (Figure 1D, expanded trace). These electrical events strongly resemble complex spikes observed in mammalian Purkinje cells, which are initiated by synaptic input from inferior olive neurons via climbing fibers (see below) (D’Angelo et al., 2011). The pattern of regular, spontaneous firing of simple spikes interspersed with occasional complex spike-like events was also observed at 6 and 7 dpf (Figures 1E,F) and was stable through 14 dpf, the last time point that was investigated (data not shown). Our results indicate that the excitability of zebrafish Purkinje cells develops within 24–48 h after the cells are born resulting in a stable firing pattern that strongly resembles the electrical activity of mammalian Purkinje cells.

**Figure 2:**
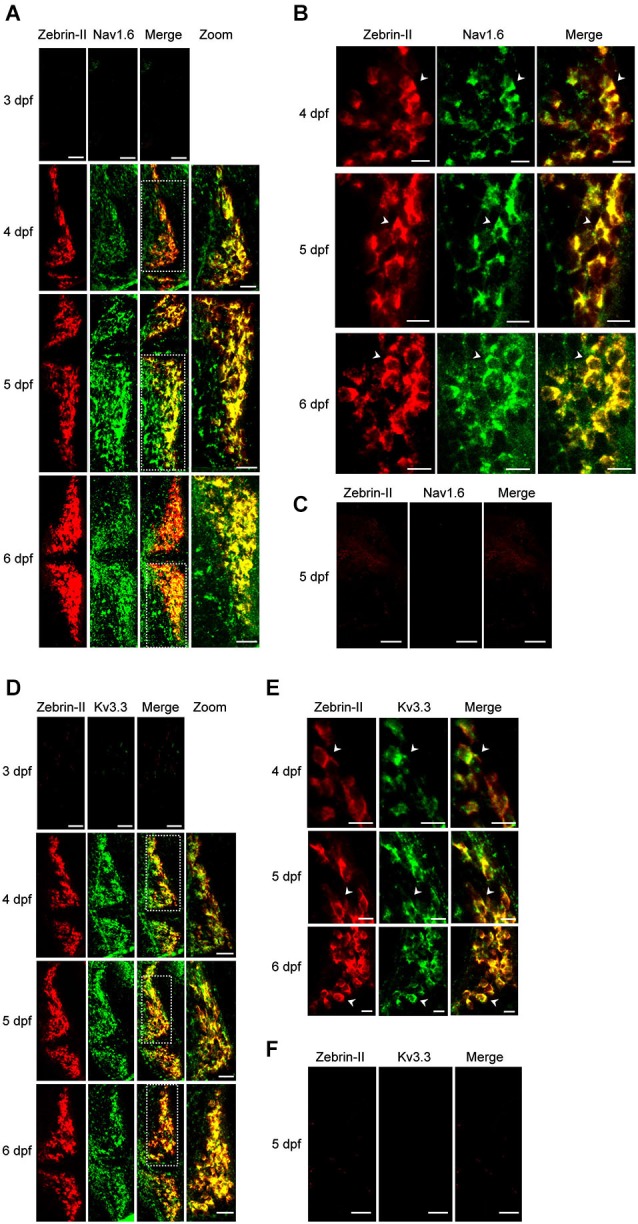
**Expression of Nav1.6 and Kv3.3 coincides with the emergence of spontaneous tonic firing in Purkinje cells. (A)** Whole mount double fluorescent *in situ* hybridization was performed on TLN zebrafish at 3, 4, 5, and 6 dpf using digoxigenin-labeled *aldoca* (Zebrin-II, red) and fluorescein-labeled *scn8aa* (Nav1.6, green) antisense probes (Thisse and Thisse, 2008; Brend and Holley, 2009). Projected confocal image stacks for *aldoca*, *scn8aa*, and merged images are shown in the 3 left-most panels, respectively. Dashed white boxes in merged images denote regions of one cerebellar hemisphere that have been enlarged and shown in the right-most panel (zoom). Here and in subsequent parts of the figure, dorsal views are shown. Anterior is to the left. Scale bars: 40 μm. **(B)** Enlarged single optical sections from the projections in Figure 2A at 4, 5, and 6 dpf are shown. Arrowheads identify individual Purkinje cell bodies. Scale bars: 10 μm. **(C)** Whole mount double fluorescent *in situ* hybridization was performed on TLN zebrafish at 5 dpf using digoxigenin-labeled *aldoca* (Zebrin-II, red, left panel) and fluorescein-labeled *scn8aa* (Nav1.6, green, center panel) sense probes (Thisse and Thisse, 2008; Brend and Holley, 2009). Merged image is shown in right panel. Scale bar: 40 μm. **(D)** Whole mount double fluorescent *in situ* hybridization was performed on TLN zebrafish at 3, 4, 5, and 6 dpf using digoxigenin-labeled *aldoca* (Zebrin-II, red) and fluorescein-labeled *kcnc3a* (Kv3.3, green) antisense probes (Thisse and Thisse, 2008; Brend and Holley, 2009). Projected confocal image stacks for *aldoca*, *kcnc3a*, and merged images are shown in the 3 left-most panels, respectively. Dashed white boxes in merged images denote regions of one cerebellar hemisphere that have been enlarged and shown in the right-most panel (zoom). Scale bars: 40 μm. **(E)** Enlarged single optical sections from the projections in Figure 2D at 4, 5, and 6 dpf are shown. Arrowheads identify individual Purkinje cell bodies. Scale bars: 10 μm. **(F)** Whole mount double fluorescent *in situ* hybridization was performed on TLN zebrafish at 5 dpf using digoxigenin-labeled *aldoca* (Zebrin-II, red, left panel) and fluorescein-labeled *kcnc3a* (Kv3.3, green, center panel) sense probes (Thisse and Thisse, 2008; Brend and Holley, 2009). Merged image is shown in right panel. Scale bar: 40 μm.

In mammalian Purkinje cells, spontaneous tonic firing arises from the interplay of resurgent Na^+^ currents, mediated in large part by Nav1.6, and rapidly activating and deactivating K^+^ currents conducted by members of the Kv3 family, particularly Kv3.3 and Kv3.4 (Raman et al., 1997; Khaliq et al., 2003; Martina et al., 2003; Akemann and Knöpfel, 2006). We used *in situ* hybridization to investigate whether Nav1.6 and Kv3.3 are expressed in zebrafish Purkinje cells during the development of spontaneous tonic firing (Brend and Holley, 2009). Differentiated Purkinje cells were identified using a probe directed against the *aldoca* (zebrin-II) gene (Tanabe et al., 2010). Expression of Nav1.6, Kv3.3, and zebrin-II was not detected in TLN animals at 3 dpf, when Purkinje cells are born (Figures 2A–F). However, expression of both channel types and zebrin-II was detected by antisense probes starting at 4 dpf (Figures 2A–F). The intensity of staining for Nav1.6 increased noticeably between 4 and 5 dpf (Figures 2A,B). These results show that the time course of Kv3.3 and Nav1.6 expression is strongly correlated with the emergence of excitability and the development of regular tonic firing, suggesting that the ion channels that underlie spontaneous pacemaking activity are conserved in zebrafish and mammalian Purkinje cells.

### Rapid development and maturation of functional connectivity in the zebrafish cerebellar circuit

**Figure 3:**
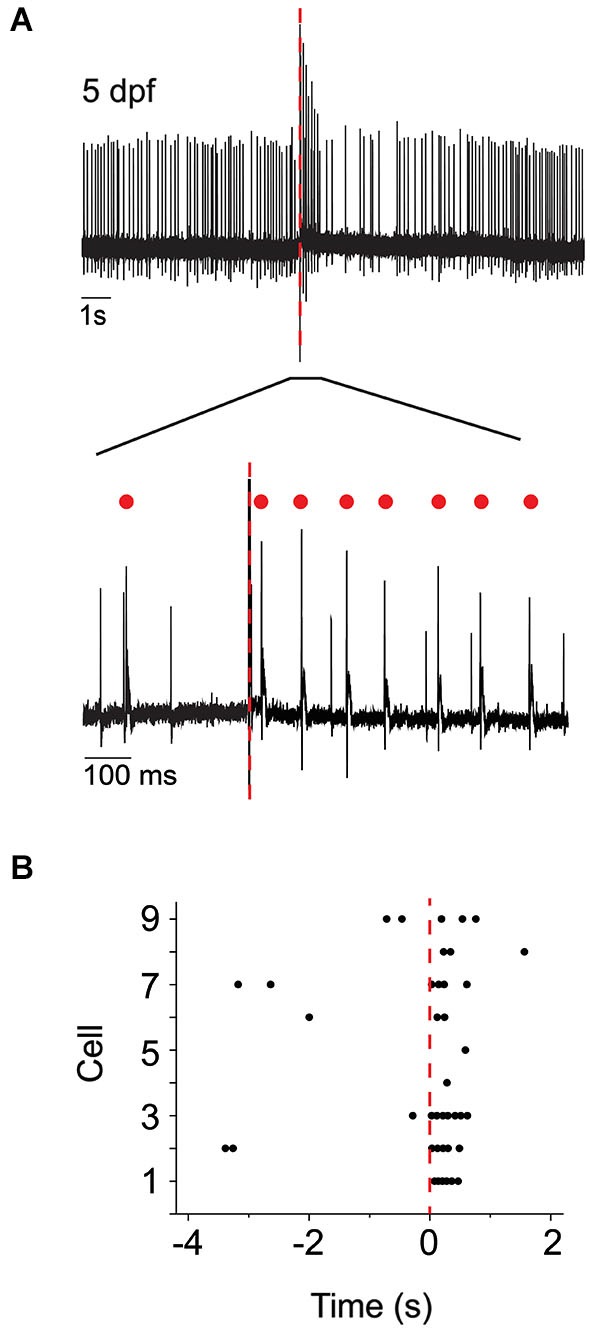
**Direct electrical stimulation of inferior olive increases occurrence of complex spike-like events. (A)** Upper: The inferior olive was stimulated using a theta pipette (see Figure 1B) at the time indicated by the red dashed line. Shown is a representative recording of Purkinje cell activity obtained at 5 dpf. Lower: The portion of the upper trace marked by a bar is shown on an expanded time scale. Red dots indicate complex spikes. **(B)** Peristimulus raster plot shows the occurrence of complex spikes before and after stimulation of the inferior olive at time 0 (red dashed line). Results were obtained from nine Purkinje cells from four animals at 5 dpf. The trace shown in panel **(A)** corresponds to cell #3.

To test the hypothesis that complex spike-like events indeed reflect the postsynaptic response of Purkinje cells to climbing fiber input, we investigated whether direct stimulation of the inferior olive increased the frequency of these events. At 5 dpf, brief supra-threshold stimuli were applied to the inferior olive using a theta pipette (Figure 1B). In the immediate post-stimulus period, the frequency of action potentials with complex waveforms in Purkinje cells increased dramatically (Figure 3). These results indicate that olivary neurons make functional climbing fiber synapses onto Purkinje cells by 5 dpf, and that activation of these synapses elicits complex spikes that strongly resemble those recorded in mammalian Purkinje cells.

**Figure 4:**
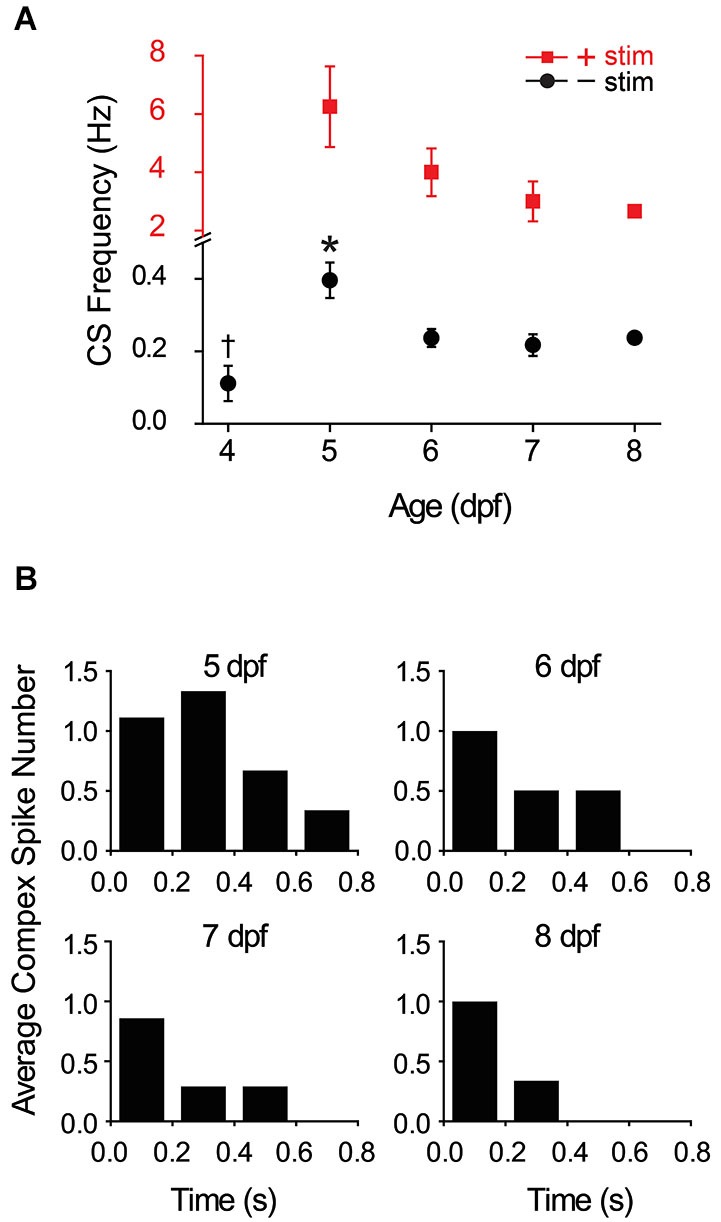
**Development and winnowing of functional connections between climbing fibers and Purkinje cells. (A)** The average frequency of complex spiking in Purkinje cells in the absence (black circles) and presence (red squares) of direct electrical stimulation of the inferior olive has been plotted vs. age. Note change in vertical scale for data obtained in the absence (black) and presence (red) of stimulation. In the absence of stimulation, complex spike frequency was averaged over 10 s recordings; the frequency at 5 dpf (0.4 Hz) was significantly higher than on all subsequent days (*, ANOVA: 4–8 dpf, *F*_(4,67)_ = 6.17, *p* = 2.75 × 10^−4^, followed by Tukey’s *post hoc* test: *p* < 0.05; *n* = 6–20 cells, 5–17 animals). The frequency at 4 dpf (0.11 Hz) was significantly lower than that measured at 6 dpf (0.24 Hz) (†, ANOVA: 4–8 dpf, *F*_(4,67)_ = 6.17, *p* = 2.75 × 10^−4^, followed by Tukey’s *post hoc* text: *p* < 0.05; *n* = 7 and 20 cells, 6 and 14 animals at 4 and 6 dpf, respectively). Complex spike frequency did not vary significantly between 6 and 8 dpf (ANOVA: 4–8 dpf, *F*_(4,67)_ = 6.17, *p* = 2.75 × 10^−4^, followed by Holm-Bonferroni *post hoc* test: 6–8 dpf, *p* ≥ 0.05; *n* = 6–20 cells, 5–14 animals). In the presence of stimulation, the frequency of complex spiking was averaged over the first 500 ms after the stimulus. Evoked complex spike frequency decreased from 6.3 Hz at 5 dpf (*n* = 9 cells, 4 animals) to 2.7 Hz at 8 dpf (*n* = 3 cells, 3 animals) (Spearman’s rank correlation, *r* = −1). Electrical stimulation increased complex spike frequency by ~16-fold at 5–6 dpf and by ~12-fold at 7–8 dpf. **(B)** Complex spiking was evoked by direct electrical stimulation of the inferior olive at 5–8 dpf. The average number of complex spikes per 200 ms bin after the stimulus has been plotted (*n* = 3–9 cells, 3–4 animals). Cells that did not fire complex spikes in the absence of stimulation were excluded from the analysis.

To investigate the development of functional climbing fiber synapses, we determined the prevalence of complex spiking as a function of developmental age in the absence of olivary neuron stimulation. At 4 dpf, complex spikes were detected in ~35% of Purkinje cells (7/19 cells). This value was not increased by direct stimulation of olivary neurons (not shown). In contrast, complex spiking occurred in ~65–70% of cells at 5–6 dpf (20/29 and 20/31 cells at 5 and 6 dpf, respectively), and in ~85% of cells at 7 dpf (19/22 cells). In cells that exhibited complex spiking, the frequency of complex spikes was low at 4 dpf, suggesting that active climbing fiber connections were just beginning to form (Figure 4A). Complex spike frequency increased dramatically at 5 dpf, attaining a maximum value of 0.40, before subsiding to an intermediate frequency of 0.24 at 6 dpf that did not differ significantly from that measured on subsequent days.

During the development of the mammalian cerebellum in the early postnatal period, individual Purkinje cells are initially innervated by multiple climbing fibers. Over the next 2 weeks, these inputs are winnowed by activity-dependent competition until each Purkinje cell is innervated by a single “winner” climbing fiber (Crepel, 1982; Bosman and Konnerth, 2009; Hashimoto and Kano, 2013). The finding that the frequency of complex spiking reaches a maximum at 5 dpf and subsequently declines to a stable level raises the possibility that zebrafish Purkinje cells are innervated by multiple climbing fibers at 5 dpf and that these are winnowed to a single input between 5–7 dpf. To test this hypothesis, the inferior olive was directly stimulated to activate all available climbing fibers. Electrical stimulation increased complex spike frequency dramatically between 5 and 8 dpf compared to the unstimulated values (Figure 4A). With or without direct stimulation, the highest frequency of complex spiking was observed at 5 dpf (Figure 4A).

Before activity-dependent winnowing is complete in mammals, individual Purkinje cells are innervated by climbing fiber inputs of differing strengths (reviewed by Hashimoto and Kano, 2013). The winner climbing fiber corresponds to the strongest input. Therefore, winnowing should be accompanied by an overall acceleration in the time course of the Purkinje cell response because strong synaptic inputs result in a more rapid approach to threshold than weaker inputs. To investigate whether zebrafish Purkinje cells are innervated by climbing fibers of different strengths at 5 dpf, we determined the average number of complex spikes occurring in sequential 200 ms bins following the stimulus at 5–8 dpf (Figure 4B). We found that the temporal distribution of postsynaptic responses changed as a function of developmental age. There was a greater number of complex spikes occurring at later times post stimulus at 5 dpf than on subsequent days (Figure 4B). Taken together, the data suggest that climbing fibers begin to make functional connections with Purkinje cells starting at ~4 dpf, that Purkinje cells are innervated by multiple climbing fibers of varying strengths at 5 dpf, and that winnowing of redundant climbing fiber inputs occurs between 5–7 dpf and is completed by ~7 dpf.

### Sensory stimulation alters Purkinje cell activity starting at 4 dpf

**Figure 5:**
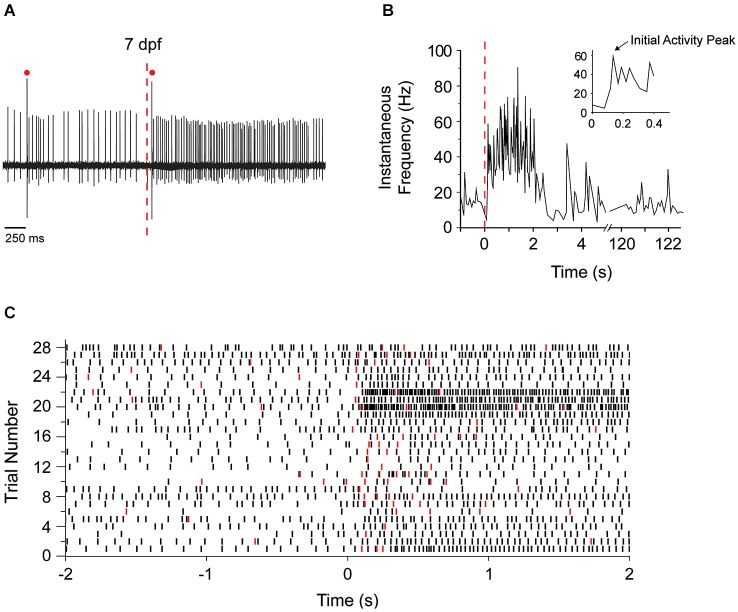
**Sudden darkness increases the frequency of tonic firing and complex spiking. (A)** Purkinje cell firing at 7 dpf was recorded before and after turning off the LED at the time indicated by the red dashed line. A representative trace is shown. Red dots indicate complex spikes. **(B)** The instantaneous tonic firing frequency in the cell shown in panel **(A)** was measured before and after the LED was turned off at time 0 (dashed red line). The frequency, which fluctuated around 15 Hz in the light, increased significantly to ~60 Hz during the first 150 ms after turning the LED off and remained elevated at ~40–60 Hz for several seconds. Inset shows the instantaneous firing frequency during the first 500 ms of darkness on an expanded time scale. The arrow indicates the initial peak in firing frequency after the stimulus, which corresponds to the direct response of the Purkinje cell to sudden darkness (see Figure 7). **(C)** Peristimulus raster plot shows the timing of simple action potentials (black symbols) and complex spikes (red symbols) before and after the LED was turned off at time 0. Data were obtained from 28 trials in 11 Purkinje cells from nine animals at 7 dpf. The trace shown in panel **(A)** corresponds to trial #23. At 7 dpf, the spontaneous tonic firing frequency in the absence of stimulation ranged from 4.5–19.5 Hz (mean ± SEM: 8.8 ± 0.98 Hz, *n* = 22 cells from 20 animals). After sudden darkness, the peak tonic firing frequency ranged from 19.4–100.7 Hz (mean ± SEM: 40.4 ± 7.4 Hz, *n* = 11 cells, nine animals).

A significant advantage of zebrafish is that the activity of cerebellar Purkinje cells can be recorded *in situ* in living, awake animals using a minimally-disturbed preparation with intact sensory systems, and afferent and efferent pathways. We took advantage of this to investigate the development of functional sensory input to the zebrafish cerebellum and its consequences for electrical activity in Purkinje cells. We chose visual stimulation because the visual system is functional at this stage of development, reproducible visual stimuli can be readily applied, and vision is highly relevant to zebrafish behavior because it guides prey capture (feeding) and contributes to the avoidance of predators (Fleisch and Neuhauss, 2006; Westphal and O’Malley, 2013; Chhetri et al., 2014). Zebrafish were adapted to a white LED light for 2 min. The visual system was then stimulated by turning the light off while recording simultaneously from Purkinje cells. Figure 5A shows the response of an individual Purkinje cell at 7 dpf to sudden darkness. Turning off the LED increased the instantaneous tonic firing frequency, from ~15 Hz prior to the stimulus to ~60 Hz in the first 150 ms after the stimulus (Figure 5B). Increases in tonic firing frequency are likely mediated by mossy fiber pathways that convey visual information to granule cells, leading to activation of parallel fiber synapses onto Purkinje cells (D’Angelo et al., 2011). Most Purkinje cells responded to the stimulus by increasing the rate of firing (Figure 5C). However, in rare instances, the firing frequency was unaltered or even decreased (Figure 5C, trials 28 and 9 respectively). This could reflect lack of visual input to the cell or an alternative wiring pattern, respectively. In addition to the increase in tonic firing frequency, the lights off stimulus transiently increased the frequency of complex spiking in the majority of Purkinje cells (Figure 5C). The similarity of the responses in most Purkinje cells may reflect the fact that the bulk of our recordings were made in a restricted region of the cerebellum.

**Figure 6:**
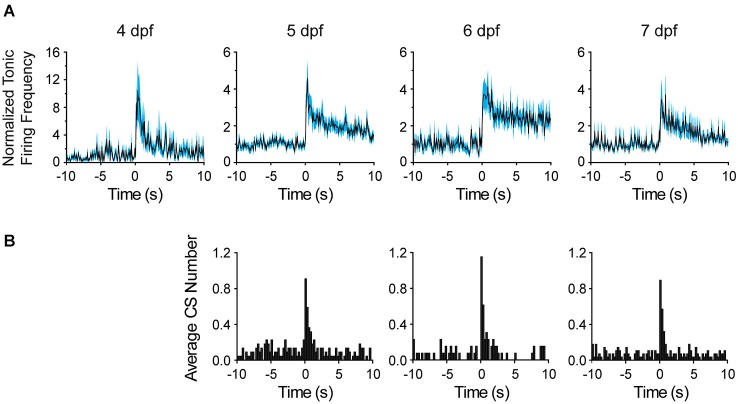
**Comparison of Purkinje cell responses to sudden darkness at 4–7 dpf. (A)** After turning the LED off at time 0, the frequency of tonic firing was calculated for each 100 ms interval and normalized to the average frequency calculated over the 10 s period prior to the stimulus (solid lines, *n* = 6–14 cells, 5–11 animals). The cyan-shaded areas represent the SEM. Please note compressed vertical scale at 4 dpf. **(B)** The bar graphs show the average number of complex spikes recorded in each 250 ms interval, calculated by dividing the total number of complex spikes in all cells by the number of cells (*n* = 13–28 trials from 8–11 cells, 5–9 animals). The LED was turned off at time 0. Note that sudden darkness did not evoke complex spikes at 4 dpf.

**Figure 7:**
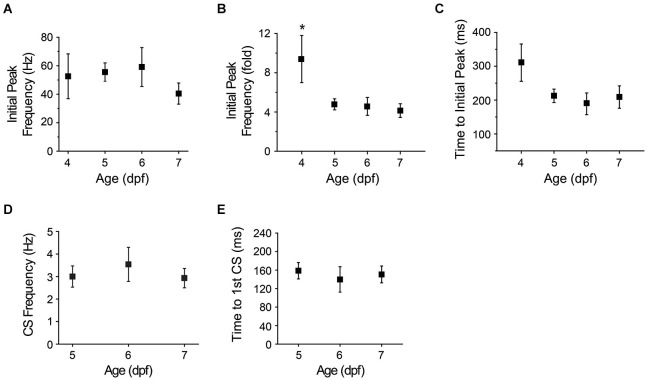
**Maturation of afferent pathways conveying visual information to cerebellum. (A)** The graph shows the initial peak in instantaneous simple spike frequency after turning off the LED at different ages (see Figure 5B, inset). Values obtained at 4–7 dpf did not differ significantly (ANOVA: 4–7 dpf, *F*_(3,35)_ = 0.75, *p* = 0.53, *n* = 6–14 cells, 5–11 animals). **(B)** Initial peak simple spike frequencies evoked by sudden darkness in panel **(A)** were normalized to the average simple spike frequency measured before turning off the LED and plotted vs. age. The fold-change in initial peak frequency was significantly higher at 4 dpf than on subsequent days (*, ANOVA: 4–7 dpf, *F*_(3,35)_ = 4.36, *p* = 0.01, followed by Tukey’s *post hoc* test: *p* < 0.05, *n* = 6–14 cells, 5–11 animals). **(C)** The latency between turning off the LED and the initial peak in simple spike frequency has been plotted vs. age. The latency decreased from 311 ± 55 ms at 4 dpf to 212 ± 19 ms at 5 dpf, 189 ± 32 ms at 6 dpf and 209 ± 33 ms at 7 dpf (*n* = 6, 14, 9 and 11 cells at 4, 5, 6 and 7 dpf, respectively, 5–11 animals; Spearman’s rank correlation, *r* = −0.8). **(D)** The graph shows the complex spike frequency measured in the first 500 ms after turning off the LED at 5–7 dpf (*n* = 13–28 trials from 8–10 cells, 5–9 animals). Values did not differ significantly. **(E)** The graph shows the latency between turning off the LED and the first complex spike at 5–7 dpf. Values did not differ significantly (*n* = 9–24 trials from 8–10 cells, 5–9 animals). Trials in which sudden darkness did not evoke complex spikes within 500 ms post-stimulation were excluded from the analysis.

To investigate the development of functional sensory input pathways to the cerebellum, we repeated the lights off stimulation experiment at different dpf. The average frequency of tonic firing after the stimulus was calculated for each 100 ms interval and normalized to the average frequency prior to the stimulus (Figure 6A). Strikingly, even at 4 dpf, turning the LED off dramatically increased the frequency of tonic spiking in the post-stimulus period. After an initial, dramatic increase in frequency, the firing rate remained elevated by ~1.5-fold over the pre-stimulus value for tens of seconds. These results indicate that functional sensory input conveyed via parallel fibers emerges within 24 h of the birth of Purkinje cells. Similarly, at 5, 6, and 7 dpf, turning the LED off transiently and dramatically increased the frequency of tonic firing. As at 4 dpf, this was followed by a persistent, ~1.4- to 2.3-fold elevation in firing rate that subsided to the baseline level over ~20–30 s.

To investigate when climbing fibers become responsive to visual input, the number of complex spikes per 250 ms intervals before and after the stimulus was plotted on peristimulus time histograms (Figure 6B). No increase in complex spiking was observed at 4 dpf (not shown), when functional climbing fiber connections are rare and complex spike frequency is low (see Figure 4A). In contrast, at 5, 6, and 7 dpf, the number of complex spikes was significantly elevated for ~1 s following the stimulus, indicating that visual input activated olivary neurons, which provide error correction signals to Purkinje cells (D’Angelo et al., 2011).

In response to sudden darkness, the tonic firing rate remained elevated for tens of seconds after the increase in complex spiking had subsided (Figure 6). This suggests that parallel fiber inputs, which innervate inhibitory interneurons in the molecular layer in addition to Purkinje cells (D’Angelo et al., 2011), maintain a net excitatory drive to Purkinje cells that is not directly evoked by sudden darkness. Therefore, additional afferent pathways and/or feedback loops are likely to contribute to the prolonged alteration in tonic firing frequency. Alternatively, climbing fiber input may result in a persistent mode switch in tonically-firing Purkinje cells (Loewenstein et al., 2005, 2006; Schonewille et al., 2006). However, this would not explain the results obtained at 4 dpf when visual stimulation did not evoke complex spikes.

The initial phase of the response to sudden darkness is presumably mediated by the pathway that connects retinal photoreceptors to Purkinje cells with the fewest synapses. To characterize the development and maturation of this pathway, we determined whether the initial response to sudden darkness varied as a function of developmental age. We identified the first peak in instantaneous tonic firing frequency in the immediate post-stimulus period (see Figure 5B, inset). Interestingly, this initial peak firing rate did not differ significantly between 4 and 7 dpf (Figure 7A). When normalized to the pre-stimulus frequency, the most dramatic increase in instantaneous firing frequency was observed at 4 dpf (10-fold) compared to subsequent days (4-fold), at least in part because the basal firing rate is significantly lower at 4 dpf compared to subsequent days (Figure 7B; see Figure 1F). We also measured the time that elapsed between the stimulus and the initial peak in tonic firing frequency, and found that the latency of the response, which was ~300 ms at 4 dpf, declined to ~200 ms at 5 dpf (Figure 7C). This suggests that the circuit reacts more promptly as it matures, which may reflect refinement of the afferent pathway circuitry.

As noted above (Figure 6B), complex spiking increased significantly in the post-stimulus period starting at 5 dpf. Similarly to the increase in the tonic firing frequency (Figure 7A), the increased frequency of complex spiking, averaged over the first 500 ms post-stimulus, did not differ significantly between 5 and 7 dpf (Figure 7D). Compared to the pre-stimulus value, complex spike frequency increased in the post-stimulus period by 8-fold at 5 dpf and ~14 fold at 6 and 7 dpf, reflecting the higher frequency of complex spiking at 5 dpf compared to subsequent days (see Figure 4A). The latency to the first complex spike post-stimulus did not differ significantly between 5 and 7 dpf (Figure 7E). These results indicate that climbing fibers respond to afferent visual input within 48 h of the birth of Purkinje cells and that the response is stable on subsequent days.

## Discussion

### Rapid functional development and highly conserved electrical properties in zebrafish cerebellum

A functional cerebellar circuit receiving afferent sensory information develops rapidly in zebrafish. Purkinje cells, which are born at 3 dpf, spontaneously fire action potentials by 4 dpf. The firing rate is modulated by visual input, likely conveyed by mossy fibers to cerebellar granule cells, which make parallel fiber synapses onto Purkinje cells (Bae et al., 2009; D’Angelo et al., 2011). Within 48 h of their birth, Purkinje cells display spontaneous tonic firing interspersed with complex spikes, which correspond to the post-synaptic response to climbing fiber activation (D’Angelo et al., 2011). Soon after they form, climbing fiber inputs respond to visual stimulation. The development of regular tonic firing in zebrafish is correlated with the time course of expression of the voltage-gated Nav1.6 and Kv3.3 channels, which underlie spontaneous pacemaking in mammalian Purkinje cells (Raman et al., 1997; Khaliq et al., 2003; Martina et al., 2003; Akemann and Knöpfel, 2006). In addition, Kv3.3 channels control the complex spike waveform (Hurlock et al., 2008; Zagha et al., 2008; Veys et al., 2013). Our results suggest that the electrical properties of mammalian and zebrafish Purkinje cells are highly conserved.

The rapid emergence of Purkinje cell excitability and cerebellar circuit activity is temporally correlated with the anatomical development of the zebrafish cerebellum (Bae et al., 2009; Tanabe et al., 2010). In zebrafish, the tri-lamellar structure of the cerebellar cortex forms by 5 dpf. The rapid time course makes zebrafish advantageous for *in vivo* studies of cerebellar development and function. In contrast, in rats, cerebellar cortical layers are not evident and Purkinje cells are not functionally mature until ~2–3 postnatal weeks, which corresponds to ~5–6 weeks post-fertilization (Altman and Bayer, 1997; McKay and Turner, 2005).

### Synapse elimination in the developing zebrafish cerebellum

Our data suggest that zebrafish Purkinje cells, like mammalian Purkinje cells, are initially innervated by multiple climbing fibers that are winnowed until only the strongest input remains (Crepel, 1982; Bae et al., 2009; Hashimoto and Kano, 2013). The frequency of complex spikes evoked by electrical stimulation of the inferior olive was significantly higher at 5 dpf than at 7 dpf. We suggest that Purkinje cells are innervated by multiple climbing fibers at 5 dpf and that these inputs are winnowed to a single input by ~7 dpf. However, our data do not rule out an alternative, non-mutually exclusive hypothesis, that an age-dependent decrease in the excitability of olivary neurons underlies the decline in complex spike frequency after 5 dpf. Nevertheless, our results indicate that reduced climbing fiber excitability is unlikely to be solely responsible. First, the number of complex spikes evoked in the first 200 ms after electrically stimulating the inferior olive did not differ significantly between 5 and 8 dpf. In contrast, a significantly higher number of complex spikes was evoked between 200 and 400 ms post-stimulus at 5 dpf compared to subsequent days (see Figure 4B). This suggests that strong and weak climbing fiber inputs co-exist on Purkinje cells at 5 dpf. Strong inputs should elicit rapid responses. In contrast, the response to weaker inputs would be delayed because weaker stimuli result in a slower approach to threshold in the postsynaptic cell. The selective loss of delayed complex spiking after 5 dpf is consistent with the specific elimination of weaker climbing fiber inputs. In contrast, if the decrease in complex spike frequency were primarily due to reduced excitability of olivary neurons, a reduction in the rate of complex spiking should manifest itself equally between 0–200 and 200–400 ms post-stimulus. Second, whereas the frequency of complex spiking evoked by direct electrical stimulation was greater at 5 dpf than on subsequent days, this was not the case when complex spiking was evoked by sudden darkness (see Figure 7D). After sudden darkness, there was no significant difference in complex spike frequency at 5, 6, or 7 dpf. The most plausible interpretation of these results is that direct electrical stimulation of olivary neurons activated both strong and weak climbing fiber inputs at 5 dpf, whereas sudden darkness, a more physiological stimulus, activated only the strong inputs. Consistent with this, the increase in complex spike frequency in response to the visual stimulus was not as great as that seen in response to direct electrical stimulation of the inferior olive (compare Figures 4A, 7D).

### Rapid development of afferent pathways conveying visual information to cerebellum in zebrafish

The zebrafish visual system is fully functional by 80 h post-fertilization (~3.3 dpf) as indicated by the presence of an active optokinetic response (Chhetri et al., 2014). We used sudden darkness as a stimulus to investigate the functional development of pathways that convey visual information to the cerebellum and to determine the effect of visual input on electrical activity in Purkinje cells. At 4 dpf, sudden darkness robustly increased the frequency of spontaneous firing, indicating that functional connections had already been made by mossy fibers onto granule cells and by granule cells onto Purkinje cells, even though Purkinje cells had not yet established a mature pattern of activity. Purkinje cells responded to sudden darkness more rapidly at 5 dpf than at 4 dpf, indicating that one or more circuit components continued to mature and undergo synaptic refinement. During this period, active synaptogenesis occurs between retinal ganglion cells and their targets in the optic tectum (Niell et al., 2004; Meyer and Smith, 2006). In addition, synapses between Purkinje cells and their presynaptic partners mature anatomically as dendritic spines form (Bae et al., 2009; Tanabe et al., 2010).

In contrast, sudden darkness did not evoke complex spiking at 4 dpf. Our data indicate that functional connectivity between climbing fibers and Purkinje cells started to emerge at 4 dpf, although the fraction of cells firing complex spikes and complex spike frequency were low. Therefore, an effect of sudden darkness on complex spiking rate might have gone undetected at 4 dpf. Alternatively, functional afferent connections conveying visual information to the inferior olive may not have developed by 4 dpf. On subsequent days, sudden darkness increased the complex spike frequency in most Purkinje cells. Interestingly, the increased rate of complex spiking lasted less than 1 s, whereas tonic firing frequency remained elevated over baseline for tens of seconds. This persistent increase is unlikely to be a direct response to sudden darkness. Rather, it may reflect feedback from other brain regions such as the thalamus, which receives input from both the visual system and eurydendroid cells, which convey efferent information from the cerebellum (Burrill and Easter, 1994; Heap et al., 2013).

Although we recorded dramatic changes in Purkinje cell activity in response to sudden darkness, we are unable to interpret the results in behavioral terms because the animals were paralyzed during electrophysiological experiments. Indeed, we cannot rule out the possibility that details of the Purkinje cell response, such as its time course, were affected by the inability of paralyzed animals to mount a behavioral response. The response of freely-behaving zebrafish at 6–7 dpf to large decreases in light intensity has been characterized by Burgess and Granato (2007). Darkness elicits large angle turns (O-bends) with novel kinematics and a latency of 200–300 ms that are distinct from the escape response (Burgess and Granato, 2007). Whether the cerebellum is involved in this behavior is unknown.

The rapid development of the cerebellum and its afferent and efferent pathways likely confers an essential survival advantage for zebrafish, which develop outside the body of the mother and must escape predators and hunt for food starting early in life (Westphal and O’Malley, 2013). However, little is known about the role of the cerebellum in controlling behavior in zebrafish. Interestingly, the functional maturation of the cerebellum between 4 and 7 dpf coincides with the time zebrafish must begin to actively hunt for food, since the yolk sac is depleted at 4–5 dpf (Westphal and O’Malley, 2013). Finding food involves prey capture, a complex, visually-guided behavior that may require cerebellar input. The emergence of this behavior is temporally well-correlated with the development of a functional cerebellum responsive to visual input. Zebrafish are incapable of capturing prey at 3 dpf, but can successfully capture Paramecia at 5 dpf (Westphal and O’Malley, 2013). Further work will be required to determine whether this behavior is under cerebellar control.

### Advantages of zebrafish for functional mapping of afferent and efferent pathways to cerebellum and for investigating cerebellar control of motor behavior

Our data indicate that zebrafish is an excellent model system in which to combine optogenetics and electrophysiological recording to map functional afferent and efferent connections to the cerebellum and to investigate cerebellar control of behavior. The electrical activity of Purkinje cells can be readily recorded *in situ* in live zebrafish with intact sensory modalities and motor output. Such experiments would be technically challenging and significantly more invasive in mice. As new genetically-encoded sensors of Ca^2+^ or voltage emerge, it will be feasible to correlate optical signals with the underlying electrical events recorded electrophysiologically at high temporal resolution *in vivo*. Such experiments are essential for interpreting optical measures of neuronal function during brain mapping experiments. In addition, our results set the stage for genetically manipulating the electrical activity of Purkinje cells in verifiable ways and determining the behavioral consequences.

### Author’s contributions

Diane M. Papazian, Jui-Yi Hsieh, Brittany Ulrich, and Jijun Wan designed research; Jui-Yi Hsieh and Brittany Ulrich performed research; Fadi A. Issa generated unpublished research tool; Jui-Yi Hsieh, Brittany Ulrich, and Diane M. Papazian analyzed data; Jui-Yi Hsieh and Diane M. Papazian wrote the paper.

## Conflict of interest statement

The authors declare that the research was conducted in the absence of any commercial or financial relationships that could be construed as a potential conflict of interest.

## Footnotes

^1^zfin.org/ZDB-GENO-080307-1
